# Editorial: Use of biostimulants in beneficial plant-microbe interactions

**DOI:** 10.3389/fpls.2025.1592681

**Published:** 2025-04-01

**Authors:** Sofia I. A. Pereira, Ricardo Aroca, Pablo Cornejo

**Affiliations:** ^1^ Universidade Católica Portuguesa, CBQF - Centro de Biotecnologia e Química Fina – Laboratório Associado, Escola Superior de Biotecnologia, Porto, Portugal; ^2^ Department of Soil and Plant Microbiology, Estación Experimental del Zaidín (CSIC), Granada, Spain; ^3^ Centro de Estudios Avanzados en Fruticultura (CEAF), Rengo, Chile; ^4^ Centro Tecnológico de Suelos y Cultivos (CTSyC), Facultad de Ciencias Agrarias, Universidad de Talca, Talca, Chile

**Keywords:** biostimulants, biofertilizers, crops, plant-microbe interactions, soil microbial communities

In recent years, the agricultural sector has faced increasing challenges due to climate change, soil degradation, and the excessive use of synthetic fertilizers and pesticides (Dedieu and Prados, 2022). As a result, there is a growing interest in sustainable alternatives that enhance crop resilience while maintaining high productivity. In this scenario, the use of biostimulants has emerged as a promising approach to address these challenges by enhancing these beneficial interactions. Another critical aspect of biostimulants’ impact is their role in reducing environmental footprints by optimizing nutrient use efficiency, topics that are covered in the current Research Topic. Beneficial microorganisms, such as plant growth-promoting rhizobacteria (PGPR), arbuscular mycorrhizal fungi (AMF), and free-living fungi, play a vital role in enhancing plant growth, improving nutrient uptake, and increasing tolerance to abiotic and biotic stresses (Lopes et al., 2021). In addition to microbial inoculants, bioactive molecules from natural sources further amplify plant benefits. Among them, seaweed extracts are particularly noteworthy, as they contain bioactive compounds such as auxins, cytokinins, and betaines, which enhance root development and improve stress tolerance (Mughunth et al., 2024; Kumar et al., 2024). Additionally, plant secondary metabolites like flavonoids play a crucial role in modulating plant-microbe interactions and fostering crop growth (Shah et al.).

This Research Topic brings together a series of studies that provide valuable insights into the role of biostimulants in enhancing plant nutrient uptake, alleviating environmental stressors, and fostering beneficial plant-microbe interactions. One of the most significant and widely recognized applications of biostimulants is their ability to enhance crop resilience against a range of abiotic stresses, including drought, salinity, extreme temperatures, and nutrient deficiencies. One study within this research topic investigates the potential of the halotolerant endophyte *Pseudomonas stutzeri* ISE12 in improving salt stress tolerance in two genotypes of *Sorghum bicolor* L. - one salt-tolerant and the other salt-sensitive (Rajabi-Dehnavi et al.). Bacterial inoculation significantly enhanced seed germination, seedling vigor, and overall plant growth in both sorghum genotypes under saline conditions. Such biostimulant effects were particularly marked in the salt-sensitive genotype, suggesting their tailored application could enhance crop resilience in varying conditions. This enhancement was achieved through the induction of antioxidant enzyme activity to mitigate oxidative stress and the accumulation of osmolytes, which help maintain cell turgor under salt stress. Shah et al. investigated the effects of flavonoids and cell-free supernatant from *Devosia* sp. - SL43, a novel bacterial strain isolated from *Amphecarpaea bracteate* root nodules, on canola and soybean growth under optimal and saline conditions. Flavonoid foliar application significantly improved soybean growth in optimal conditions but had no impact under salt stress. In contrast, the cell-free supernatant effectively mitigated salt stress, increasing soybean growth. Canola showed a weaker overall response, except for root parameters, which were significantly improved by flavonoid treatment. While flavonoids improved soybean growth in optimal conditions, cell-free supernatants mitigated salt stress effects, emphasizing the combined potential of biostimulants for sustainable agriculture. Flavonoids have also recently gained significant attention for their role in modulating plant-AMF interactions by acting as signaling molecules during the pre-symbiotic phase, enhancing spore germination and colonization of tomato roots (Lidoy et al.).

In recent decades, the use of these bio-based solutions has garnered significant attention for their ability to enhance nutrient uptake and promote crop growth while reducing the reliance on chemical fertilizers. Altimira et al. evaluated the potential of *Bacillus safensis* RGM 2450 and *B. siamensis* RGM 2529 to boost *Solanum lycopersicum* yield while reducing chemical fertilizer use. Inoculated plants achieved higher yields and enhanced rhizosphere colonization, particularly by *Flavobacterium* strains, improving soil microbial diversity. This approach enabled a 33% reduction in chemical inputs without compromising productivity.

Biostimulants also strengthen plant defenses, helping to mitigate biotic stressors such as pests and diseases. Among them, *Trichoderma* spp. is particularly valued for promoting plant growth and suppressing disease. Recent research demonstrated that *Sclerotium rolfsii* stress negatively affects peanut seedling growth, but inoculation with *Trichoderma harzianum* QT20045 significantly mitigates this effect. Strain QT20045 enhances plant growth by increasing ACC deaminase activity and indole-3-acetic acid production, both crucial for stress tolerance and development (Wang et al.). Hu et al. conducted a bibliometric analysis on research trends related to medicinal plant microbiomes, highlighting the role of microorganisms in plant growth, pest control, and secondary metabolite production. The study emphasizes the importance of understanding microbial communities in plants, particularly in the context of biostimulants and their potential applications in sustainable agriculture and medicinal plant production. Additionally, the research shows that the field of biostimulants is continuously growing, with a notable presence in countries such as India, China, and Brazil. Biostimulants influence plant-microbe interactions, altering the abundance and composition of active compounds in medicinal plants, thus demonstrating their potential for product development from medicinal plants.

The impact of biostimulants, such as seaweed extract and a vegetal-derived protein hydrolysate, on *Escherichia coli* population dynamics in leafy crops like lettuce is a critical food safety concern. Understanding their influence on pathogen abundance on plant surfaces is essential for ensuring consumer health and regulatory compliance. Fiore et al. found that while seaweed extract and a vegetal-derived protein hydrolysate promote plant-associated aerobic bacteria, they simultaneously reduce *E. coli* viability on lettuce leaves. These results highlight biostimulants as a potential sustainable strategy to enhance the microbiological quality of ready-to-eat leafy greens.

In summary, the studies compiled in this Research Topic provide compelling evidence that biostimulants are a sustainable and effective strategy for enhancing crop yield and resilience. By applying microbial inoculants and plant-derived compounds to plants and soil, farmers can improve stress tolerance, optimize nutrient uptake and boost crop productivity, while reducing reliance on synthetic inputs. Furthermore, biostimulants strengthen plant defense mechanisms and suppress foliar pathogens, contributing to improving crop health and food safety. However, further research is essential to optimize biostimulant application, further elucidate their mechanisms of action, and evaluate their long-term impact across various crops and soil types.
